# Supplementary material for: Haplotype-aware diplotyping from noisy long reads
Source: Genome Biol. 2019 Jun 3;20:116. doi: 10.1186/s13059-019-1709-0 (PMC6547545; doi:10.1186/s13059-019-1709-0)
Supplement: Supplementary file 1 — We provide precision, recall, and genotype concordance of MarginPhase on PacBio and WhatsHap on Nanopore reads. Furthermore, we give the results of cutting and downsampling Nanopore reads. We also provide more detailed phasing statistics and report results on re-typing the GIAB indels. Additionally, we show how precision, recall, and F-measure of our callsets behave as a function of read depth and analyze homozygous and heterozygous calls separately. We also provide figures that show the genotyping performance of our methods for different thresholds on the reported genotype qualities. (PDF 695 kb) [file 13059_2019_1709_MOESM1_ESM.pdf]

Haplotype-aware genotyping from noisy long reads - Supplementary material

Jana Ebler, Marina Haukness, Trevor Pesout, Tobias Marschall, Benedict Paten

1 Comparison Against High Confidence Truthset

In Figure S1 we provide a comparison against the GIAB high confidence truthset (within high confidence regions) [1]. In the main manuscript, we present the more performant method for each sequencing technology; here we describe the results for the less performant method.

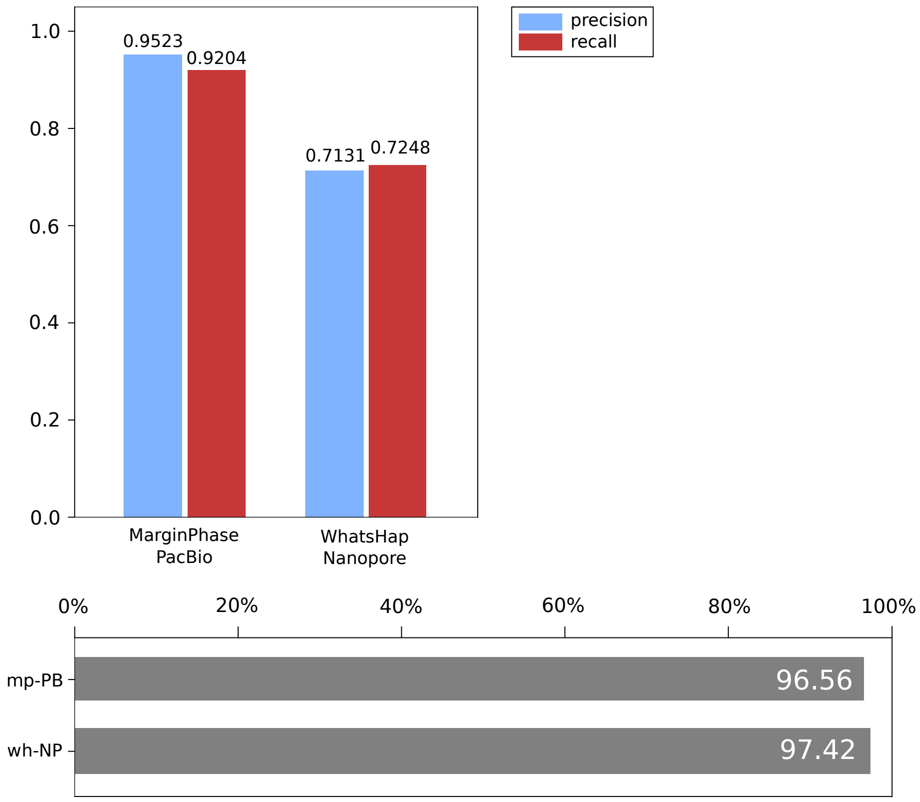

Figure S1: ***Precision and Recall*** *(Top) of MarginPhase on PacBio and WhatsHap on Nanopore data sets in GIAB high confidence regions.* ***Genotype Concordance (Bottom)*** *(wrt. GIAB high confidence calls) of MarginPhase (mp, top) on PacBio and WhatsHap (wh, bottom) on Nanopore.*

2 Cutting and Downsampling Reads

In Figure S2, we show how the genotyping error behaves as a function of coverage for different lengths of provided read fragments. In the main manuscript, we present the results for the PacBio data, here we give corresponding results for the Nanopore reads. As we observed previously, the genotyping error increases, as the length of the reads decreases due to the lack of information on neighboring variants.

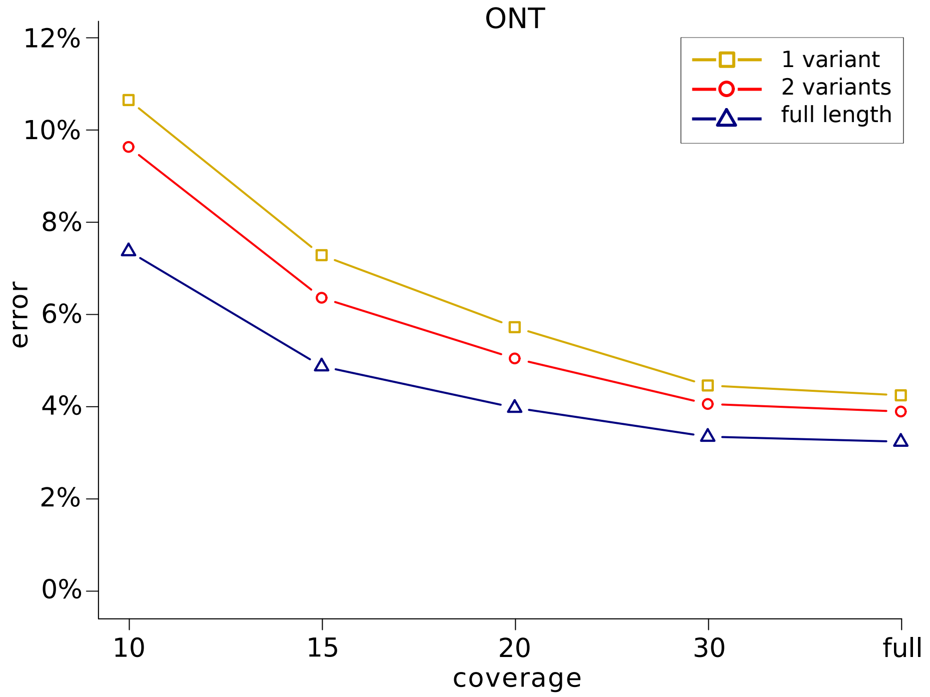

Figure S2: ***Genotyping Errors*** *(wrt. to GIAB calls) as a function of coverage. The full length reads were used for genotyping (blue) and additionally, reads were cut such as to cover at most two variants (red) and one variant (yellow).*

3 Switch Error Rates (inside of high confidence blocks)

In Table S1 we describe the switch error rates of our methods for the two sequencing technologies within the GIAB high confidence regions [1]. For the computation of switch errors, we only consider variant positions genotyped as heterozygous in both the callset and ground truth.

**chromosome**	**MP-PB**	**WH-PB**	**MP-NP**	**WH-NP**
chr1	0.48%	0.37%	0.32%	0.65%
chr10	0.43%	0.37%	0.27%	0.63%
chr11	0.20%	0.03%	0.07%	0.41%
chr12	0.25%	0.03%	0.09%	0.45%
chr13	0.23%	0.02%	0.07%	0.37%
chr14	0.18%	0.02%	0.06%	0.38%
chr15	0.22%	0.02%	0.09%	0.35%
chr16	0.55%	0.55%	0.45%	1.01%
chr17	0.65%	0.69%	0.54%	1.34%
chr18	0.25%	0.04%	0.09%	0.35%
chr19	0.11%	0.06%	0.21%	1.42%
chr2	0.21%	0.02%	0.08%	0.37%
chr20	0.24%	0.06%	0.15%	0.54%
chr21	0.18%	0.02%	0.07%	0.43%
chr22	0.55%	0.61%	0.45%	1.12%
chr3	0.27%	0.06%	0.11%	0.34%
chr4	0.18%	0.01%	0.06%	0.20%
chr5	0.21%	0.01%	0.09%	0.33%
chr6	0.79%	0.75%	0.53%	0.85%
chr7	0.32%	0.19%	0.19%	0.53%
chr8	0.26%	0.09%	0.10%	0.36%
chr9	0.18%	0.01%	0.07%	0.43%
chrX	0.32%	0.04%	0.12%	0.23%
whole genome	0.32%	0.17%	0.17%	0.50%

Table S1: *Switch error rates of MarginPhase and WhatsHap for each chromosome inside of the GIAB high confidence regions.*

4 Results for Indels

Since WhatsHap and MarginPhase currently cannot detect indels, we re-genotyped the GIAB truth set variants using the WhatsHap implementation (as WhatHap is able to re-genotype given variant positions). We computed the genotype concordance for indels by determining the fraction of correctly genotyped positions among all positions in the truth set for which a genotype could be computed. We also report which fraction of the variants could not be genotyped by our method either due to the position being multi-allelic or because no genotyping information is available at that site after WhatsHaps allele detection and readselection steps. The results are shown in Table 4.

	**genotype concordance**	**not genotyped**
indels (PacBio)	73.82%	6.82%
indels (Nanopore)	55.98%	7.38%

Table S2: *Results from re-typing GIAB truth set indels using WhatsHap on the PacBio and Nanopore reads.*

5 Read Depth Analysis

In Fig S3 and S4 we provide an analysis of precision, recall, and f-measure (the harmonic mean of the precision and recall) for our method as a function of read depth. To produce this data, we analyzed our calls with `rtg vcfeval` [2] against the GIAB benchmark small variant calls v3.3.2 [1] in their high confidence region. We annotated the outputted true positive, false positive, and false negative VCF files with the read depth at each variant's reference locus. For each read depth, we counted the number of TP, FP, and FN calls and used them to derive accuracy statistics.

For each sequencing technology and method implementation we plot three pieces of data: in dotted lines, the precision and recall for the calls made at that specific read depth; in solid lines, the precision and recall for all calls made at or above the read depth; and in grey, the amount of calls which were made at each read depth. The vertical line indicates the maximum f-measure considering all variants found at that depth or above. Maximum plotted depth is 100 for PacBio and 75 for ONT; these values were selected as they slightly surpass twice the median depth of the BAMs (46$\times$ and 37$\times$ respectively).

As is apparent from the plots, the precision and recall are varied at lower depths (less than 20), and at higher depths (roughly 1.5$\times$ the median depth), and that these correlate with areas where fewer calls were made. We hypothesize that the decreased accuracies at higher depth are related to copy number variation in the sample.

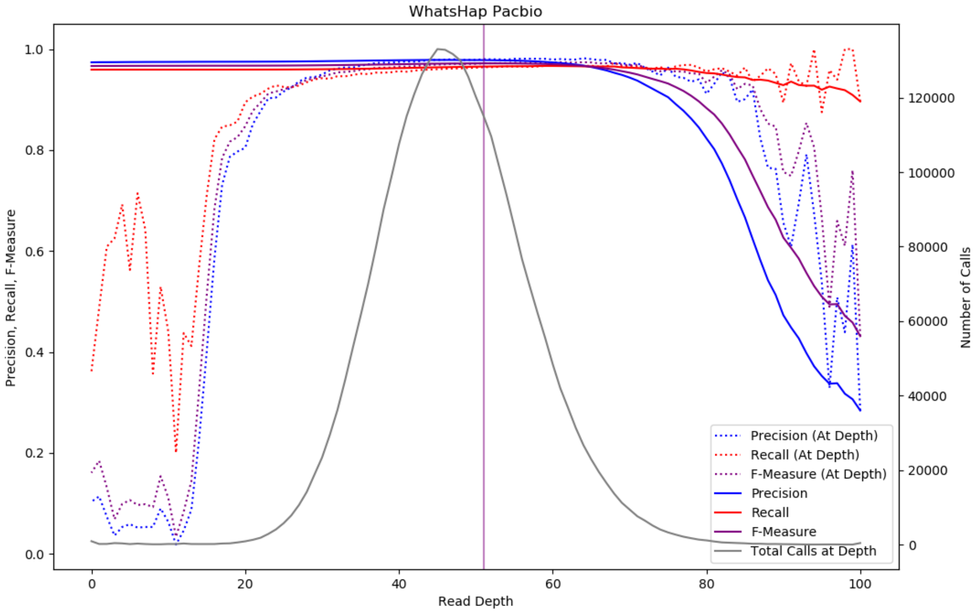

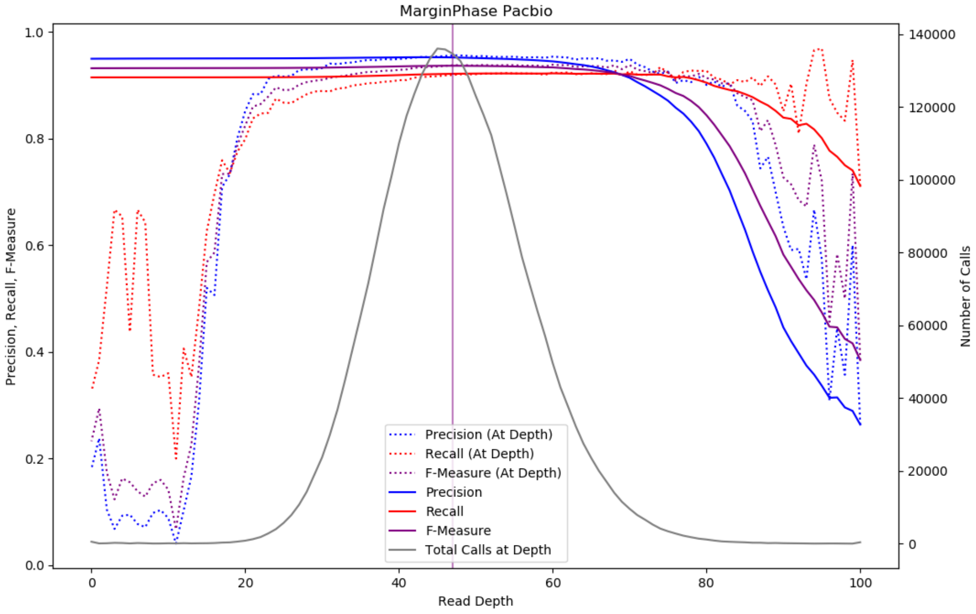

Figure S3: ***Read Depth: PacBio*** *Precision, Recall, and F-Measure as a function of depth.*

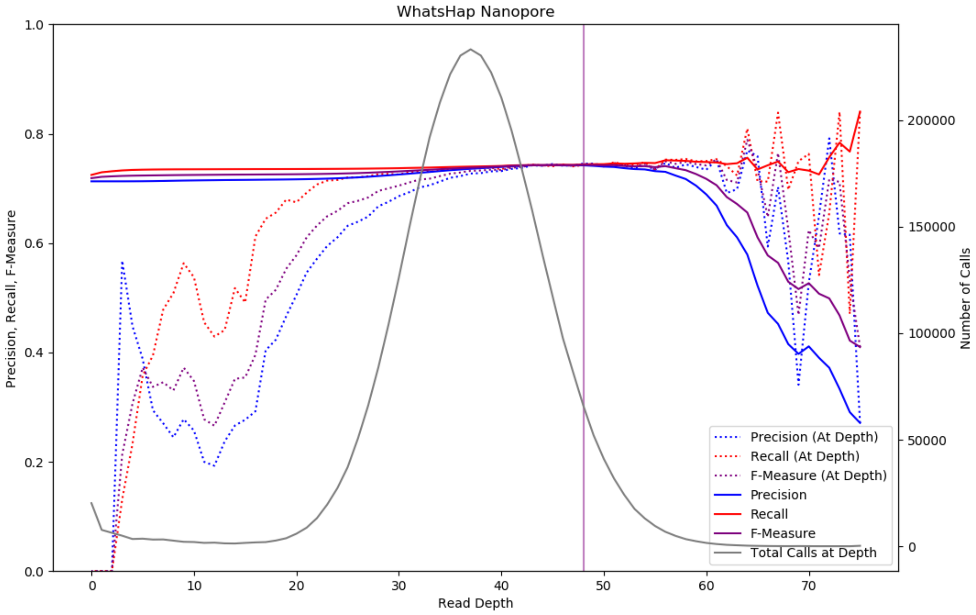

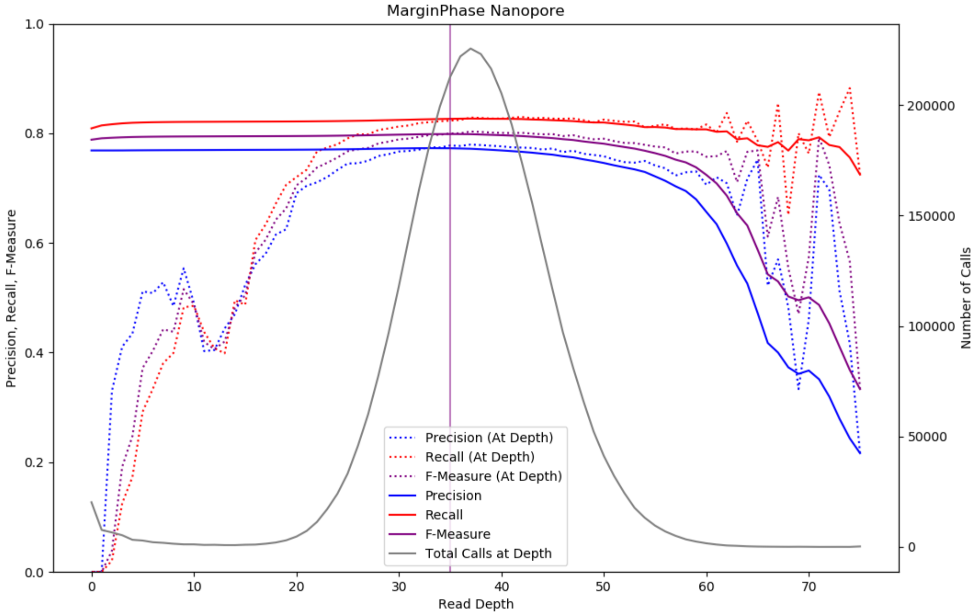

Figure S4: ***Read Depth: Nanopore*** *Precision, Recall, and F-Measure as a function of depth.*

6 Genotyping Results on Heterozygous Variants vs Homozygous Variants

We present our genotyping results on the Genome in a Bottle truth set in high confidence regions, splitting up the performance on variants that were heterozygous and variants that were homozygous alternate in the truth set. The results are shown in Tables S3 and S4. In summary, the precision is better at homozygous sites for all cases (using both tools MarginPhase and WhatsHap, on both PacBio and Nanopore reads). Recall is also better at homozygous sites in most cases, except in the MarginPhase-PacBio run, where it is worse. The difference in performance between heterozygous and homozygous sites is quite drastic when nanopore sequencing is used, especially in regards to precision. Perhaps this means that the programs are predicting many more false variants due to the distribution of errors seen in highly inaccurate reads, and the error models are not yet tuned well enough to take that into account.

	**Precision**	**Recall**	**F-Measure**
WhatsHap (PacBio)	0.9827	0.9928	0.9877
WhatsHap (Nanopore)	0.9369	0.9382	0.9376
MarginPhase (PacBio)	0.9940	0.8923	0.9404
MarginPhase (Nanopore)	0.9923	0.8448	0.9126

Table S3: *Summary of genotyping results on homozygous variants.*

	**Precision**	**Recall**	**F-Measure**
WhatsHap (PacBio)	0.9678	0.9377	0.9525
WhatsHap (Nanopore)	0.5721	0.5870	0.5795
MarginPhase (PacBio)	0.9241	0.9291	0.9266
MarginPhase (Nanopore)	0.6647	0.7858	0.7202

Table S4: *Summary of genotyping results on heterozygous variants.*

7 Genotype Likelihoods

Our methods output a likelihood for each possible genotype at a variant site and makes a prediction by reporting the likeliest genotype at each position. From the genotype likelihoods, we compute the probability that the reported genotype is wrong by subtracting the likelihood of the predicted genotype from 1. Computing the corresponding phred-score of this value yields the genotype quality.

In order to analyze the reported genotype qualities, we first computed the genotyping concordance of our PacBio and Nanopore callsets with respect to the GIAB truth set as a function of the amount of genotyped variants when using different thresholds on the genotype quality (Figure S5). For each threshold value (0, 20, 50, 80, 100, 150, 200, 300, 400, 500) we considered the percentage of variants reported with a higher quality score ("variants genotyped") and computed the genotype concordance of this set of variants. Each dot in the plots represents a different threshold, in ascending order from right to left. As it can be seen in Figure S5, higher thresholds on the genotype quality lead to smaller amounts of genotyped variants. At the same time, the genotype concordance increases since many wrong, low confidence calls are removed. The maximum quality value output by MarginPhase is limited to 100. Therefore, the set of variants genotyped with higher thresholds is empty and no genotype concordance can be computed.

In a second experiment, we compared the genotype concordance of each set of calls reported with the same genotype quality to the expected genotype concordance as given by the respective qualities. Resulting plots are shown in Figure S6. The size of each dot corresponds to the number of calls that were reported with the underlying quality in the respective VCF file. Both methods reported high quality values for the majority of calls and the observed genotype concordances for these variants were close to the expected ones. However, plots show that the genotype qualities produced by WhatsHap and MarginPhase are not yet well-calibrated. We expect that improving the computation of weights that we assign to the entries of the allele matrix will lead to better quality scores as the computation of forward and backward probabilities is based on these weights (see Section 5 in the main paper).

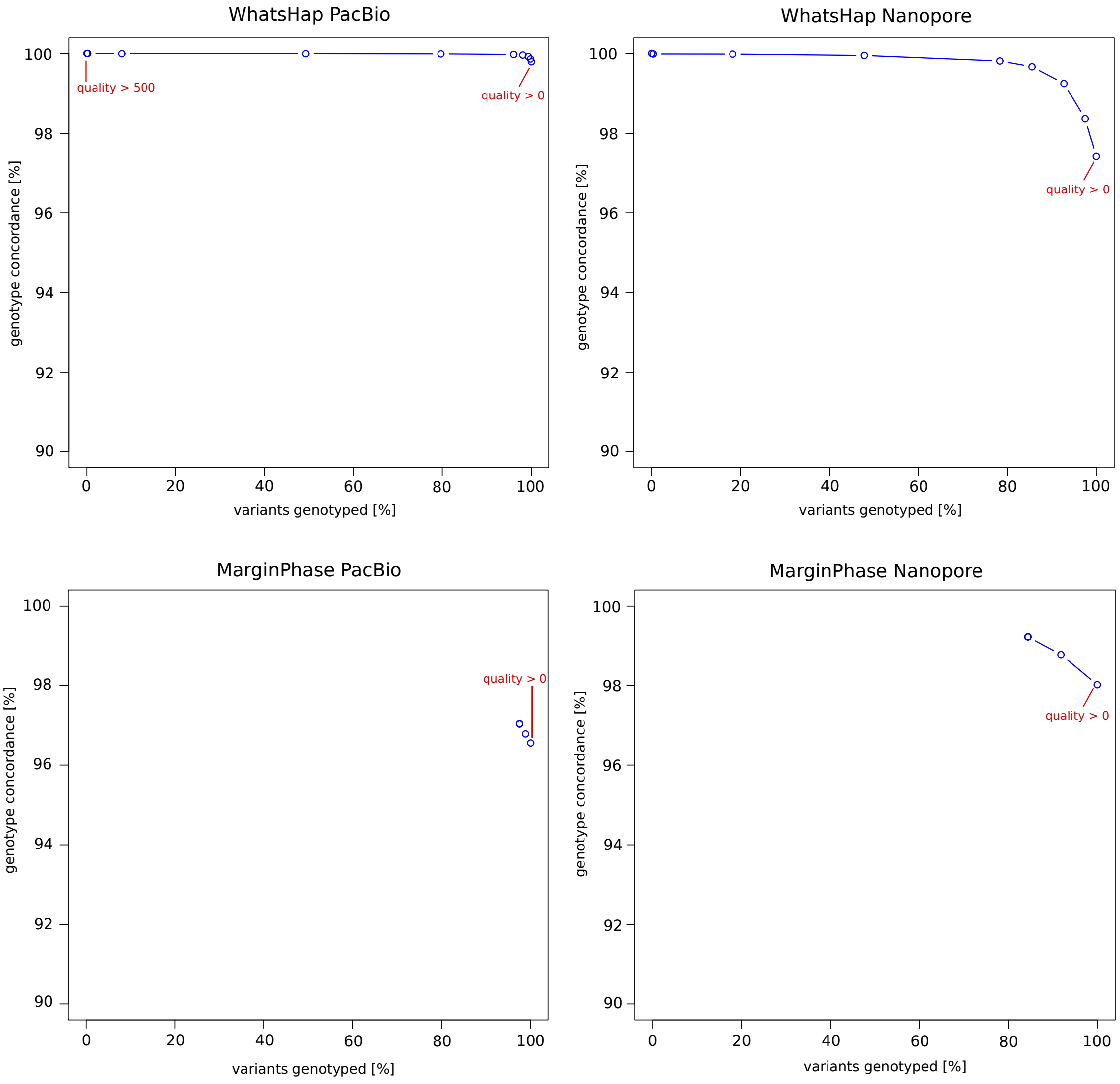

Figure S5: *Genotyping concordance as a function of genotyped variants for different thresholds (0, 20, 50, 80, 100, 150, 200, 300, 400, 500) on the genotype quality. Since the maximum quality value output by MarginPhase is limited to 100, thresholds larger than 100 are not considered in the plots.*

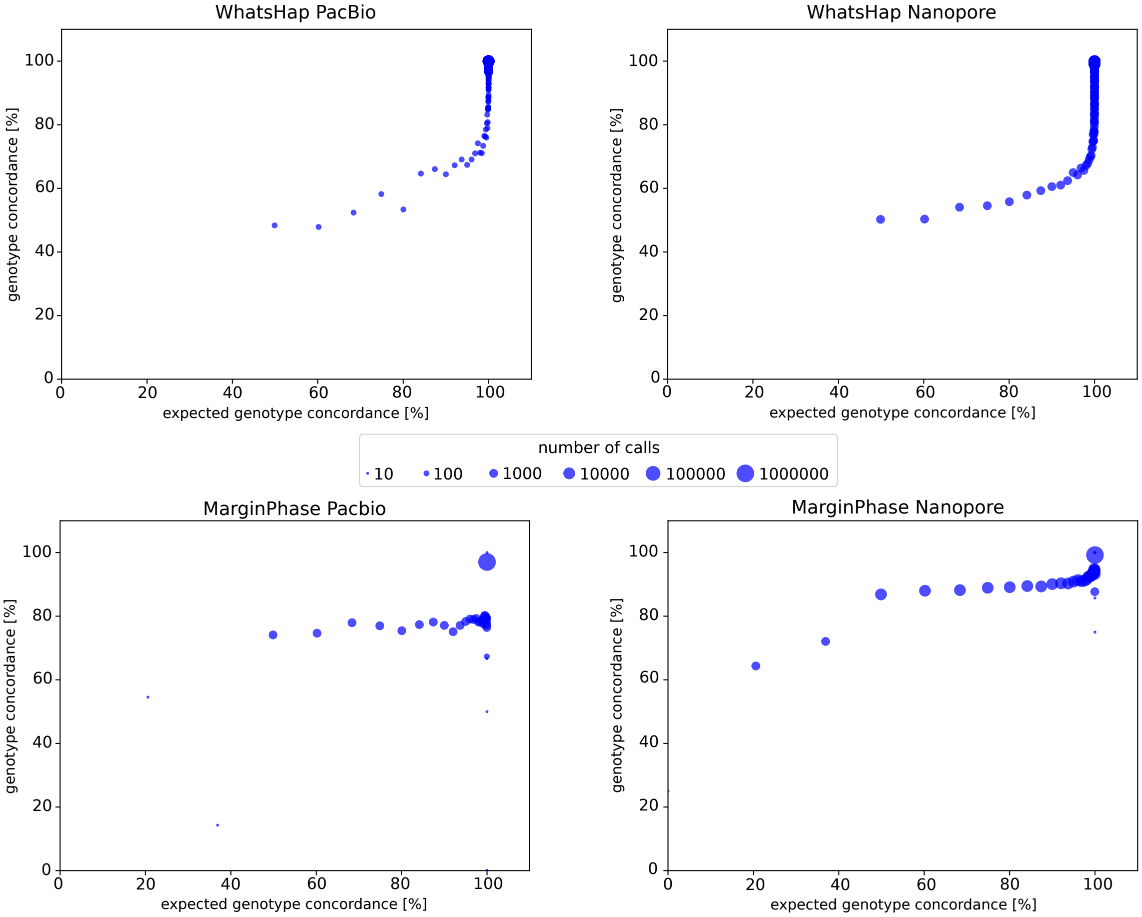

Figure S6: *Observed genotype concordance as a function of the expected genotype concordance of the variant calls. Dot sizes correspond to the number of calls which were reported with the same quality score.*

Availability of data and material

The datasets generated and analyzed during the current study as well as the version of the source code used are available at `http://doi.org/10.5281/zenodo.2616973` [3].
MarginPhase and WhatsHap are released as Open Source software under the MIT licence. MarginPhase is available at `github.com/benedictpaten/marginPhase`, WhatsHap is available at `bitbucket.org/whatshap/whatshap`.

References

[1] Zook, J., McDaniel, J., Parikh, H., Heaton, H., Irvine, S.A., Trigg, L., Truty, R., McLean, C.Y., De La Vega, F.M., Salit, M., *et al.*: Reproducible integration of multiple sequencing datasets to form high-confidence snp, indel, and reference calls for five human genome reference materials. bioRxiv (2018). doi:10.1101/281006

[2] Cleary, J.G., Braithwaite, R., Gaastra, K., Hilbush, B.S., Inglis, S., Irvine, S.A., Jackson, A., Littin, R., Rathod, M., Ware, D., *et al.*: Comparing variant call files for performance benchmarking of next-generation sequencing variant calling pipelines. bioRxiv (2015). doi:10.1101/023754

[3] Ebler, J., Haukness, M., Pesout, T., Marschall, T., Paten, B.: Haplotype-aware diplotyping from noisy long reads Data sets (2018). doi:10.5281/zenodo.2616973
